# Disorder Control in Crystalline GeSb_2_Te_4_ Using High Pressure

**DOI:** 10.1002/advs.201500117

**Published:** 2015-06-30

**Authors:** Ming Xu, Wei Zhang, Riccardo Mazzarello, Matthias Wuttig

**Affiliations:** ^1^ Institute of Physics (IA)RWTH Aachen UniversityAachen52074Germany; ^2^ Institute for Theoretical Solid State PhysicsRWTH Aachen UniversityAachen52074Germany; ^3^ JARA‐FIT and JARA‐HPCRWTH Aachen UniversityAachen52056Germany

**Keywords:** ab initio molecular dynamics (AIMD), disorder, Ge—Sb—Te (GST), high pressure, phase‐change materials

## Abstract

Electronic phase‐change memory devices take advantage of the different resistivity of two states, amorphous and crystalline, and the swift transitions between them in active phase‐change materials (PCMs). In addition to these two distinct phases, multiple resistive states can be obtained by tuning the atomic disorder in the crystalline phase with heat treatment, because the disorder can lead to the localization of the electronic states and, thus, hamper the electron transport. The goal of this work is to achieve and explore multiple disordered configurations in PCMs by applying high pressure. Large‐scale ab initio molecular dynamics simulations demonstrate that pressure can lower the energy barrier for the antisite migration in crystalline PCMs. The accumulation of these antisite atoms largely increases the compositional disorder, adding localized electronic states near the conduction band. The disorder‐induced electron localization triggered by pressure is a novel way to modulate the properties of materials. Furthermore, the random distortion of the lattice induced by the compositional disorder provides a new mechanism that contributes to the amorphization of crystalline PCMs at high pressure.

## 1 Introduction

Disorder in crystals refers to deviations from the atomic arrangement of a perfect lattice. It can be associated, for example, to the random distribution of vacancies, the alloying of different types of elements, or the occupation of antisites, e.g., sites usually reserved for other elements. The degree of disorder is often governed by thermodynamic laws. Entropy always favors disorder, driving the atomic arrangement to be as disordered as possible. However, usually such chemical disorder is discouraged by the concomitant enthalpy rise, caused by the formation of homopolar bonds. Disorder can induce localization of electronic wavefunctions (e.g., Anderson localization^1^ and thus can be crucial for the electronic properties of materials.^2^, ^3^, ^4^, ^5^, ^6^, ^7^

The current applications of phase‐change materials (PCMs) take advantage of the fast transformations and large property contrast between the amorphous and the crystalline states.^8^, ^9^, ^10^, ^11^ Such binary memory devices, however, may fail to meet the increasingly demanding requirements of data storage. A possible solution to this issue is to record data on each memory cell with multiple states of electrical resistivity, in addition to “on” and “off” switches. This may be achieved by tuning disorder in PCMs. Therefore, it is important to understand and control disorder in these materials. The development of a multistate memory device would significantly increase the data density and could change the way electronic devices work.^12^, ^13^

To manipulate the disorder in data storage media, Siegrist et al. have modified the atomic arrangement in crystalline PCMs such as Ge–Sb–Te (GST), a prototype of PCMs, by annealing.^2^ At low annealing temperatures, these GST samples form metastable cubic rocksalt phases (*c*‐GST) with different levels of electrical resistivity. In this phase, one sublattice contains Te atoms, whereas Ge, Sb, and vacancies occupy the sites of the second sublattice in a random fashion. High annealing temperatures induce the ordering of the vacancies in *c*‐GST, which gradually evolves into a metallic hexagonal phase (*h*‐GST) with all vacancies diffusing into layers. Ab initio simulations^3^ show that such multiple resistive states are indeed due to the different degrees of vacancy ordering. In particular, strong disorder results in the localization of electron wave functions at the Fermi energy.

In this article, we report that the compositional disorder in PCMs can also be tuned by pressure in lieu of the thermal treatment. Large‐scale ab initio molecular dynamics (AIMD) simulations reveal that pressure can expedite the antisite hopping in the vacancy‐ridden *c*‐GST by lowering the migration barrier. Accumulation of these antisites leads to severe atomic distortions. The resulting strong misalignment of bonds may trigger the loss of the long‐range order in the crystal^14^ and contributes to the amorphization of *c*‐GST under high pressure, as observed in experiments at 15 GPa.^15^, ^16^ Our simulations identify a new disorder‐triggered mechanism of the amorphization of *c*‐GST and open a new avenue to control the disorder, and hence the transport properties in PCMs.

## 2 Results and Discussions

### 2.1 Anomalous Cooperative Antisite Hopping Under Pressure

**Figure 1:**
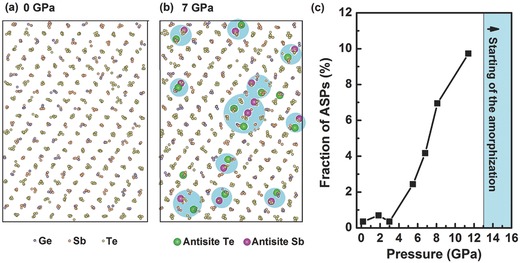
High‐pressure‐induced antisite hopping. We have performed AIMD simulations on *c*‐GST with 1008 atoms under various pressures. a,b) The atomic structures of *c*‐GST after 40 ps AIMD simulations without pressure and with moderate pressure (≈7 GPa). At moderate or high pressures, we observe a number of antisite jumps (the Te atom hops into an adjacent intrinsic vacancy site and the Sb atom then fills the empty site that the Te has left behind). The antisite Sb (Sb in Te layers) and Te (Te in Ge/Sb layers) are highlighted with green and pink spheres. The blue circles in the background mark the resulting ASPs, which result from the cooperative migration. Ge atoms are barely involved in such migration at moderate pressure. c) The percentage of ASPs (with respect to the total number of Sb atoms) increases with pressure. After 13 GPa, the crystal starts to turn into a glass.

We plot the atomic structure of *c*‐GST containing 1008 atoms with and without pressure after 100 ps of an AIMD run in **Figure** **1** (detailed structure information and computational parameters are listed in the Structure Information and Computational Methods Section). Few interlayer hopping events are observed if the lattice is subject to zero pressure (Figure 1a). In contrast, we observe significant antisite hopping by increasing the pressure to 7 GPa, as shown in Figure 1b, in which the antisite Sb and Te atoms are highlighted (the GST still maintains the cubic lattice without undergoing a phase transformation at this pressure). The large number of vacancies in Ge/Sb layers provides ample room to accommodate the adjacent Te. These antisite Te atoms are sometimes observed in the rapid crystallization from a supercooled GST liquid using AIMD simulations,^17^, ^18^ but are rarely seen in an equilibrated *c*‐GST at ambient pressure because each antisite Te creates five Te–Te homopolar bonds, which have much higher energy than the heteropolar Ge–Te and Sb–Te bonds. Under high pressure, however, Te atoms shift into the neighboring vacant sites to release the strain energy of the compressed heteropolar bonds.

Even though these Te atoms frequently hop into adjacent vacancy sites, the resulting antisite Te configurations are rather unstable. Interestingly, some of the Te atoms move back swiftly, while others are stabilized by a neighboring Sb atom, which jumps into the vacant spot the Te atom has left behind. This is the reason why antisite Sb and Te atoms always appear in pairs in Figure 1b. Unlike the single antisite hopping of Te, the resulting atomic arrangement of this cooperative antisite hopping of Sb and Te is able to be retained for quite a long time in our simulations, suggesting a possible metastable structure. Ge, on the contrary, is rarely observed to participate in any atomic migration in our simulations.

To quantify the number of antisite Sb and Te pairs (ASPs), one has to distinguish the antisite hopping from the distortion due to the thermal vibrations. We select the “cutoff” for the atomic migration in such a way that, if the movement of an atom toward the vacancy site makes all of its original bonds longer than *r*_max_ (the length of the maximum heteropolar bond when the system is subject to zero stress), this atom is then considered to enter the antisite region. *r*_max_ is located in the first minimum of the pair distribution functions (PDF) of *c*‐GST at the ambient pressure (*r*_max_ = 3.57 Å, as determined in Figure S1 in the Supporting Information). In other words, if the migration does not lead to the breaking of bonds (most of its bond lengths are shorter than *r*_max)_, then we treat this movement as a regular vibration instead of an antisite hopping. Figure 1c depicts the percentage of ASPs as a result of the cooperative hopping at elevated pressure, each derived from the instantaneous structure after 100 ps AIMD simulations. The cooperative antisite hopping rarely takes place below 5 GPa yet becomes very frequent at 7 GPa or above. After 13 GPa, the crystalline lattice is severely distorted and the system commences to amorphize. More details about ASPs as a function of pressure, temperature, and simulation time can be found in Table S1 (Supporting Information).

**Figure 2:**
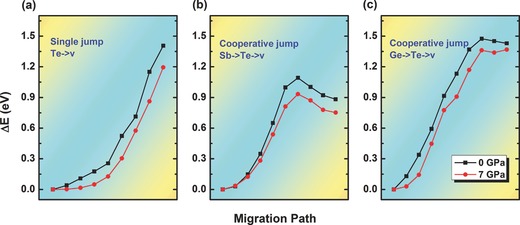
Energy barriers for three migration pathways. NEB calculations provide the insight why antisite Sb/Te pairs can form under pressure, while neither single Te hops nor the formation of Ge/Te pairs are favored. The calculations were performed both for ambient and moderate pressure (≈7 GPa) at 0 K. a) The energy barrier that a single Te climbs over when it migrates into the neighboring vacancy. The pressure can reduce the energy cost of the migration but the energy increases monotonously, so that antisite Te does not find a metastable position at moderate pressure. b) The average energy barrier for the synchronized Sb/Te hopping. Te moves toward a neighboring vacancy, while Sb moves toward the empty site that this Te has left. Such cooperative migration has an average energy barrier of 1.1 eV at zero pressure and 0.9 eV at 7 GPa. The energy basin at the end of migration path explains why ASPs are metastable. c) The energy penalty of the synchronized Ge/Te hopping is rather high, indicating the low probability of the formation of antisite Ge/Te pairs.

To gain a better understanding of this anomalous migration behavior, three questions need to be addressed: 1) why is the single hopping of Te atoms unstable? 2) Are ASPs indeed metastable? And 3) why is Ge not involved in the cooperative migration? To this end, we adopt the nudged elastic band (NEB) method^19^ to calculate the energy barriers for these three migration pathways, as shown in **Figure** **2**. The calculated energy barriers are the average results of 8–10 configurations to ensure reasonable statistics. The calculations are performed at 7 GPa as well as ambient pressure. We choose 7 GPa because at this pressure, the antisite hopping frequently takes place, while the distortion of the lattice is not very severe yet.

Figure 2a shows the energy cost of moving a single Te atom to its neighboring vacancy site. Obviously, the generation of such antisite Te requires large energies and does not lead to a metastable position. This single‐Te‐hopping scenario is rarely seen at ambient pressure, but can frequently take place at high pressure because the pressure reduces the energy cost, in particular when Te approaches the antisite, e.g., a moderate pressure of about 7 GPa can reduce the energy of the final state by 0.2–0.3 eV. Intuitively, the pressure‐induced antisite migration is a result of the increasing strain energy due to the compression of the bonds, which drives the Te atoms to move toward the area occupied by the vacancy. When the pressure is high enough, this single Te antisite hopping may become irreversible, leading to the collapse of the crystalline system.^20^

However, when Sb cooperates with Te and participates in an antisite hopping event, the energy penalty can be significantly reduced. Figure 2b shows the energy barrier of the synchronized migration of a pair of Sb and Te atoms. The pressure further reduces the energy barrier by 0.15 eV, and more interestingly, the destination at the migration path sits in an energy basin, demonstrating that this final configuration is metastable. Ge atoms are not involved in this cooperative hopping, because the energy penalty is high and the antisite Ge and Te pairs are barely metastable independent of pressure, as shown in Figure 2c. The high energy penalty originates from the large binding energy of Ge–Te bonds as compared to Sb–Te bonds.^9^

## 2.2 The Disorder‐Induced Electron Localization

**Figure 3:**
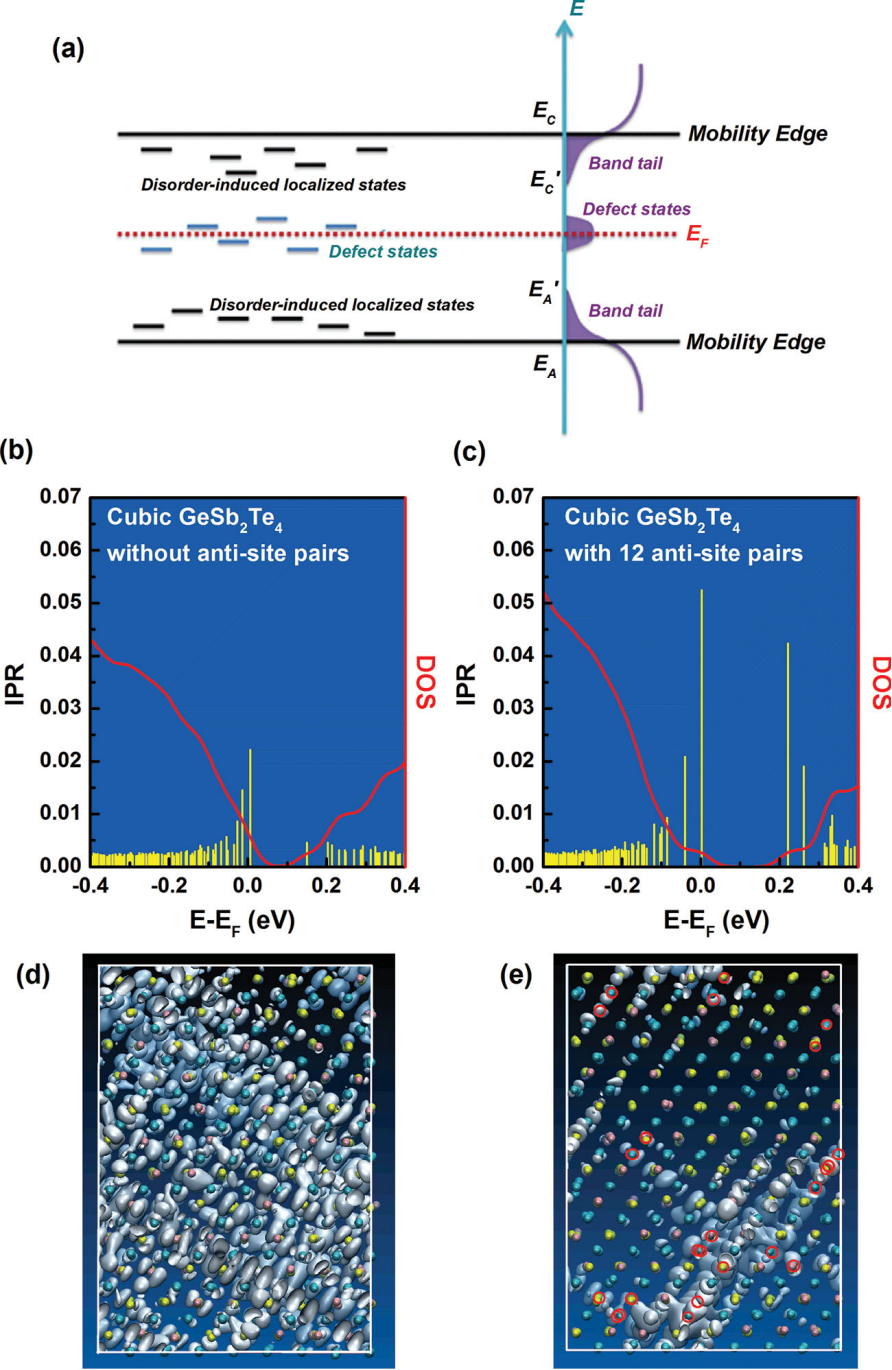
Electron localization due to the antisite hopping. a) Davis–Mott model^21^ for typical density of states of disordered semiconductors. The disorder leads to the localization of electron wavefunctions near the extended (delocalized) states. In the *c*‐GST, the Fermi energy moves toward the valence band due to the defect formation on the cation sites. b,c) The density of states and IPRs of configurations without and with ASPs. The ASPs induce a peak in IPR near the conduction band (≈0.2 eV) in (c) suggesting that electron states (≈0.2 eV) are highly localized. Both extended states (without ASPs) and localized states (with 12 ASPs) near the conduction band are projected (in white isosurfaces) onto the real‐space atomic diagrams in (d) and (e), respectively. The pink, yellow, and blue spheres in (d) and (e) denote Ge, Sb, and Te atoms, and the red circles in (e) mark the positions of ASPs. IPRs and real‐space projections reveal that the ASPs indeed cause localized states near the bottom of the conduction band. The disorder of vacancies will also lead to electronic localization near the top of the valence band with and without ASPs.^3^

The localization of electronic states often stems from compositional disorder that is introduced by defects, which entails impurity states within the band gap of semiconductors,^21^ as shown in the schematic viewgraph for the density of states of a disordered system in **Figure** **3**a. In an intrinsic semiconductor, the generation of vacancies is inevitable because they increase the entropy of the system. Consequently, the dangling bonds around these vacancies will introduce some defect states, which often fall in the band gap. However, the large fraction of stoichiometric vacancies in chalcogenides does not produce a significant amount of dangling bonds because, on average, each lattice site has three *p* electrons to maintain resonant bonding.^22^ Nevertheless, in real *c*‐GST samples, excess vacancies at cation sites are present and act as dopants. Hence, the Fermi level moves toward the valence band, rendering them p‐type semiconductors.^2^, ^12^

The ASPs can be viewed as point defects: they generate several homopolar bonds, which introduce some defect states within or near the band gap. On the other hand, ASPs add compositional disorder to the lattice, enhancing the electron localization. This disorder‐induced electron localization (Anderson localization^1^ prevails in amorphous semiconductors, but has also been observed in disordered crystalline system such as *c*‐GST,^2^ in which it is induced by the random distribution of vacancies on the sublattice. In an ordered system, the electron wavefunctions are extended periodically according to Bloch's theorem. However, disorder breaks the periodicity and results in localized states, usually located near the edges of conduction and valence bands. The borders separating the localized and delocalized states are denoted as mobility edges^23^ (see Figure 3a). The mobility edges can be moved by tuning the disorder, enabling us to modulate the band gap (or more precisely, the mobility gap) and the properties of the materials (e.g., more disorder will lead to more localized states, resulting in a larger mobility gap).

In order to quantify the degree of localization of electronic wavefunctions, we use the inverse participation ratio (IPR),^24^ to characterize how many atoms the electron wavefunctions extend to. The IPR of an electron state is roughly inversely proportional to the number of atoms that the state is distributed over. In our simulations, it is calculated as $\mathrm{IPR}(\alpha )=\sum_{i}|\varphi_{\alpha ,i}{|}^{4}/{\left(\sum_{i}^{}|\varphi_{\alpha ,i}{|}^{2}\right)}^{2}$, where $\varphi_{\alpha ,i}$ are the expansion coefficients of the Kohn–Sham eigenstates *α* with respect to the localized Gaussian‐type orbitals (GTOs) forming the basis set and *i* is counted over all the GTOs. Theoretically, the IPR of an extended state tends to zero and has a finite number for a localized state. For finite (or periodic) systems, the size of the unit cell determines the minimum value of the IPR.

Figure 3b,c shows the IPRs for two models of *c*‐GSTs with and without antisite disorder as a function of the energy of electronic states. Both structures are quenched down to 0 K and fully relaxed at 0 GPa (by increasing the box size). Since the ASPs are metastable, most of them are able to survive in such nonthermal relaxations. Figure 3b shows the IPR of *c*‐GST containing no antisite atoms, while Figure 3c represents the 100 ps AIMD configuration which was relaxed at zero pressure: the resulting configuration contains 12 ASPs in a 1008‐atom GeSb_2_Te_4_ box.

Electron localization can be observed on the top of valence band (near 0 eV) in both configurations, due to random distribution of vacancies on the sublattice.^3^ In addition, the IPR of the configuration with antisite defects (Figure 3c) shows a prominent peak at the bottom of the conduction band (near 0.2 eV), indicating that these electronic states are strongly localized (IPR = 0.043). To understand the origin of the localization, we project charge‐density isosurfaces of the lowest unoccupied molecular orbitals onto the real space (see Figure 3d,e). Isosurfaces render a value of 0.004 a.u. for both configurations. Indeed, the electron states near the bottom of the conduction band are localized around the antisite atoms. This is because the homopolar bonds (Te–Te, Ge–Sb, and Sb–Sb) promote excess electrons to the antibonding orbitals, and these defect states fail to couple with the extended states in the conduction band, resulting in their localization.^25^

### 2.3 ASP‐Triggered Unoriented Distortion and Instability of Lattice

Distortions may exist both in ordered and disordered systems, but have different forms. In ordered systems such as rhombohedral GeTe (*r*‐GeTe), where Ge and Te atoms occupy separate layers along [111] direction, the spacing between layers is not equal, one smaller and every other larger,^26^ to stabilize the lattice. This so‐called Peierls distortion results in three short bonds and three long bonds and all atoms on the same layer are uniformly distorted. However, the distortion is no longer uniform in a disordered crystal such as *c*‐GST in which Ge/Sb/vacancies are randomly distributed. Due to the random displacement of the atoms, the local structure of *c*‐GST is not as well defined as that in the ordered crystal, but the long‐range order is still maintained.

**Figure 4:**
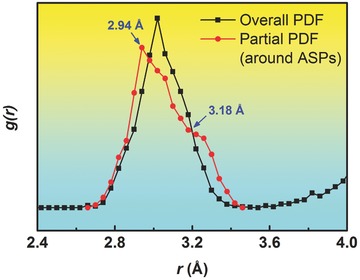
ASP‐induced random distortion in crystalline GST. To identify the distortion around ASPs, the partial PDF, which only considers the bond length around ASPs, is compared with the overall PDF. ASPs create some homopolar bonds such as Ge–Sb, Sb–Sb, and Te–Te, which have different bond lengths from heteropolar bonds. This leads to a broadened peak in the partial PDF, indicating that the bond length around the ASPs is less uniformly distributed.

When the ASPs accumulate, the lattice is expected to have even more distortions, which can be reflected on the overall PDF, *g*(r), in which the first peak denotes the bond length. The sharper the peak is, the more well defined the local structure is. On the contrary, a broadened first peak corresponds to a randomly distorted local structure. However, *c*‐GST has large disorder in the cation site and the atoms are intrinsically distorted, a small fraction (less than 10%) of ASPs may not be visible on the overall PDF. To observe the distortion caused by the antisite hopping, we devised a partial PDF, which illustrates the radial distribution of first‐nearest neighbors around ASPs. In **Figure** **4**, the comparison between the normalized overall and partial PDFs of relaxed structures at ambient pressure is displayed. The overall PDF is derived from the initial relaxed *c*‐GST configuration without ASPs, whereas the partial PDF was calculated by quenching several AIMD models (<9 GPa), which still preserve various amounts of ASPs, to zero pressure. Obviously, the partial PDF is broader than the overall one, suggesting that ASPs introduce further distortions. The first peak of the partial PDF is located around 2.94 Å, corresponding to the Ge–Sb and Sb–Sb bonds, and the shoulder near 3.18 Å is due to the longer Te–Te bonds. The distortion may be induced by the antisite atoms because the resulting homopolar bonds have different stable bond lengths than the heteropolar bonds (which is around 3.02 Å, as shown by the first peak of overall PDF in Figure 4).

Next, we manually increase the fraction of ASPs to amplify their effect. We switch 10%–50% of Sb atoms with neighboring Te and run AIMD simulations at 600 K for 15 ps. All simulations are performed at approximately zero pressure to see how the lattice evolves once it is highly disordered. Indeed, we observe the broadening of the first peak in the PDFs with increasing disorder, confirming that the disorder can cause severe local distortions. Above 50% ASPs (with respect to the number of total Sb atoms) we can barely distinguish layers anymore, and the crystal presumably starts to evolve into an amorphous system (the PDFs and atomic models are shown in Figure S2, Supporting Information). A new perspective of these results is that, the crystalline lattice is able to survive a large fraction of ASPs (≈50%), enabling us to manipulate the disorder in *c*‐GST using pressure without destroying the lattice (the observed fraction of ASPs is about 10% at the pressure of 11.4 GPa, much less than 50%, as shown in Figure 1c).

Previous ab initio work by Kolobov et al. has demonstrated that the random misalignment of the atoms in crystalline GST may trigger the loss of long‐range order.^14^ This unique mechanism of amorphization is, to the best of our knowledge, only observed in PCMs. As discussed in the present article, pressure can add compositional disorder to *c*‐GST by increasing the number of ASPs (Figure 1). This disorder leads to severe random distortions in the lattice (Figure 4). Such distortions displace the atoms off the perfect lattice points, resulting in the misalignment of them, and the crystal may eventually amorphize due to the misalignment. Pressure can accelerate this process: without applying pressure, *c*‐GST needs more than 50% ASPs to lose its long‐range order (Figure S2, Supporting Information), but with high pressure, it may require much less disorder to become amorphous. This is caused by a further reduction of the energy cost for atomic migration upon increasing pressure, which directly leads to some irreversible distortions in the lattice when the pressure is sufficiently high. For example, Caravati et al. found Te atoms hop into vacancies and fail to move back at about 14 GPa.^20^ Such irreversible atomic flips are observed in our simulations as well at higher pressure (≈11.4 GPa, see Figure S3 in the Supporting Information). The trajectories at this high pressure show that more than half of antisite Te atoms, created by single jumps (which are not stabilized by the antisite Sb), are able to persist even for a long simulation time. This suggests that permanent nonthermal distortions have been created. Hence, our ASP‐induced distortion and the irreversible atom flips at high pressure both play important roles in the amorphization of *c*‐GST for pressures approaching 15 GPa.^27^

## 3 Conclusion

To summarize, we have studied the atomic migration of *c*‐GST under various pressures using large‐scale AIMD simulations. A number of antisite hops is observed at medium pressure, with one Te atom hopping into the vacant cation site first, followed by a Sb atom filling the remaining empty Te site. This cooperative hopping has increased the compositional disorder in the crystal lattice, producing several interesting consequences for the atomic and electronic structure of *c*‐GST: 1) the atomic disorder leads to the localization of electrons. As revealed by IPR, several localized states found near the mobility gap are induced by these ASPs, in addition to those due to vacancy disorder. This interesting feature may be employed to tune the electrical properties in the memory devices using high pressure. 2) The compositional disorder can randomly distort the lattice of *c*‐GST. Such random distortion may eventually contribute to the instability of the lattice and amorphize the crystal at higher pressure.

## 4 Structure Information and Computational Methods

### 4.1 Structure of GST Polymorphs

GSTs lie on the tie‐line between GeTe and Sb_2_Te_3_ in the ternary phase diagram.^10^ Most of those materials exhibit phase‐change properties and are promising candidates for memory applications. With different fabrication methods and temperature annealing or applied pressure, GSTs can transform into various phases:

*a‐GST*: Amorphous GST can be obtained from 1) as‐sputtered film, 2) melt‐quenching the *c*‐GST, or 3) compressing the *c*‐GST to ≈15 GPa. The as‐sputtered and melt‐quenched *a*‐GST have a similar structure, in which atoms are mostly octahedral‐coordinated (*p*‐bonding) with 17% of voids (or vacant open space) percolated over the glass.^28^, ^29^ The as‐sputtered *a*‐GST is found to have more tetrahedral Ge than the melt‐quenched one.^29^, ^30^, ^31^ The high‐pressure *a*‐GST at ≈15 GPa is also dominated by the octahedral arrangement, yet the voids are largely squeezed out by pressure.^32^, ^33^

*c‐GST*: Metastable cubic GST bears a rocksalt‐like structure. Te atoms occupy one fcc sublattice while Ge/Sb/vacancies occupy the other.^34^ The fraction of vacancies relies on the stoichiometry of the (GeTe)*_x_*(Sb_2_Te_3_)_1−*x*_ compositions.^35^ In this article, we focus on cubic GeSb_2_Te_4_ which has 25% vacancies on the Ge/Sb sublattice. Te layers and Ge/Sb/vacancies layers alternately stack along [1 1 1] direction, and we build our supercell in such a way as to better evaluate the compositional disorder: *a* and *b* are two perpendicular lattice vector inside the (1 1 1) plane and *c* is oriented along the [1 1 1] direction (see Figure 1).

*h‐GST*: Stable hexagonal GST has a trigonal lattice symmetry. The major difference between *h*‐GST and *c*‐GST is that vacancies are ordered into layers in *h*‐GST.^2^ Whether Ge and Sb are mixed or occupy separate layers, however, is still under debate.

*bcc‐GST*: By compressing the above three GST phases to a pressure of 30 GPa, a body‐centered‐cubic phase can be obtained.^36^, ^37^, ^38^ All three elements are now completely mixed, forming a random solid solution. However, it is argued that *bcc*‐GST exhibits some degree of order because it can “memorize” its original phase,^39^ e.g., upon decompressing, *bcc*‐GST that is compressed from *a*‐GST and *c*‐GST will transform to the glass, but the one from *h*‐GST will go back to the original hexagonal phase.

### 4.2 Large‐Scale AIMD Simulations

AIMD simulations have been carried out by employing the “second‐generation” Car–Parrinello scheme,^40^ GGA‐PBE exchange‐correlation functionals^41^ and scalar‐relativistic Goedecker pseudopotentials,^42^ which are implemented in the CP2K suite of programs^43^ with a mixed Gaussian and plane‐wave scheme.^44^ The Kohn–Sham orbitals were expanded in a Gaussian basis set with triple‐zeta plus polarization quality, whereas the electron density was expanded in plane waves with a cutoff of 300 Ry. The AIMD simulations were performed in a canonical ensemble with a stochastic Langevin thermostat (NVT). The time step employed was 2 fs.

The rocksalt‐like GeSb_2_Te_4_ model containing 1008 atoms was built in an orthorhombic supercell, in which **a**, **b**, and **c** vectors corresponds to [−1 1 0], [−1 −1 2], and [1 1 1] directions in the cubic system. Te layers and random Ge/Sb/vacancies layers stack alternately along [1 1 1] direction (or *c*‐direction, as shown in Figure 1a). The hydrostatic pressure was generated by applying the volume reduction to the supercells, in the range of 0–10 GPa in steps of 1–2 GPa each. The AIMD simulations are performed at 300, 450, and 600 K for 60–100 ps. We mainly focused on the temperature of 600 K, because the high temperature is necessary to accelerate the migration process so that atomic hopping can be captured on a reasonable AIMD time scale. Besides, our simulation time (<100 ps) is not long enough to accomplish any first‐order diffusion‐dominant transformations, e.g., we did not observe the interplanar vacancy ordering that is necessary in the transition from the cubic to the hexagonal phase, which may take hours at 600 K in experiments. The entire simulation parameters including temperature, pressure, and simulation time are listed in Table S1 (Supporting Information). The IPR for each Kohn–Sham eigenstate has been calculated with the localized Gaussian‐type orbitals, as described in detail in ref. [3].

### 4.3 NEB Calculations

The NEB calculations^19^, ^45^ were performed at 0 K by using the Vienna ab initio simulation package code,^46^ based on the density functional theory (DFT). The projector augmented‐wave method^47^ with the GGA‐PBE^41^ for the exchange‐correlation functional was employed. Supercells containing 84 atoms (Ge_12_Sb_24_Te_48_) were built with Te occupying the anion sites, while Ge/Sb/vacancies fill the cation sites. We optimize the initial configuration (before the jump) and final configuration (after the jump), and then applied the NEB method to find the minimum energy path (MEP). Several possible transition points (“images”) are first interpolated between the initial and the final states, and the elastic band method added suppositional spring forces between neighboring images to maintain reasonable intervals between them. The calculations will converge when MEP is found. We calculated several paths for each pattern (single Te jump, cooperative jump of Sb/Te and Ge/Te), and averaged the energies to obtain Figure 2.

## Supporting information

As a service to our authors and readers, this journal provides supporting information supplied by the authors. Such materials are peer reviewed and may be re‐organized for online delivery, but are not copy‐edited or typeset. Technical support issues arising from supporting information (other than missing files) should be addressed to the authors.

Supplementary
