# Supplementary material for: Disorder Control in Crystalline GeSb2Te4 Using High Pressure
Source: Adv Sci (Weinh). 2015 Jun 30;2(8):1500117. doi: 10.1002/advs.201500117 (PMC5034799; doi:10.1002/advs.201500117)
Supplement: Supplementary file 1 — Supplementary [file ADVS-2-0k-s001.pdf]

Copyright WILEY-VCH Verlag GmbH & Co. KGaA, 69469 Weinheim, Germany, 2015.

ADVANCED SCIENCE
Open Access

# Supporting Information

for *Adv. Sci.*, DOI: 10.1002/advs.201500117

# Disorder Control in Crystalline $GeSb_2Te_4$ Using High Pressure

*Ming Xu,* Wei Zhang, Riccardo Mazzarello, and Matthias Wuttig**

Copyright WILEY-VCH Verlag GmbH & Co. KGaA, 69469 Weinheim, Germany, 2015.

# Supporting Information

for Adv. Sci., DOI: 10.1002/advs.201500117

**Disorder Control in Crystalline $GeSb_2Te_4$ using High Pressure**

*Ming Xu*, Wei Zhang, Riccardo Mazzarello, and Matthias Wuttig**

# Supporting Information

## Disorder Control in Crystalline $GeSb_2Te_4$ using High Pressure

*Ming Xu**, *Wei Zhang, Riccardo Mazzarello, and Matthias Wuttig**

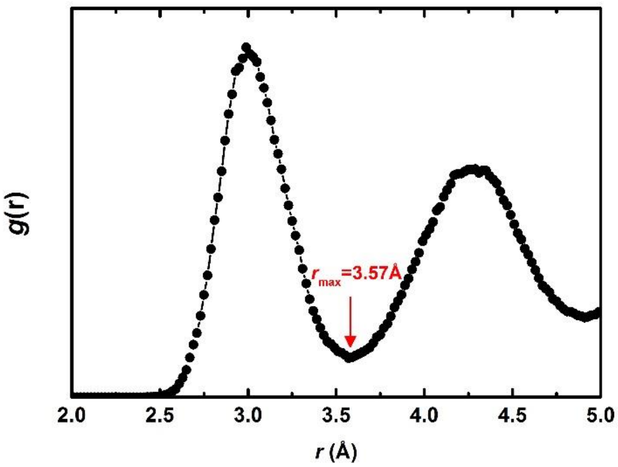


Figure S1. The PDF of *c*-GST at 600K at ambient pressure. The first minumun is the maximun heteropolar bond length ($r_{max}$=3.57Å), which is used as a "cutoff" to distinguish whether the atomic motion is an anti-site hopping or thermal vibration, e.g., if a Te atom moves towards a neighboring vacancy site by a distance that makes its original bonds (excluding the new formed bonds) longer than $r_{max}$, then it breaks the Ge-Te or Sb-Te bonds and enters the anti-site region. On the contrary, if most of the original bonds are still shorter than $r_{max}$ after the movement, then it is merely a distortion due to the thermal vibration.

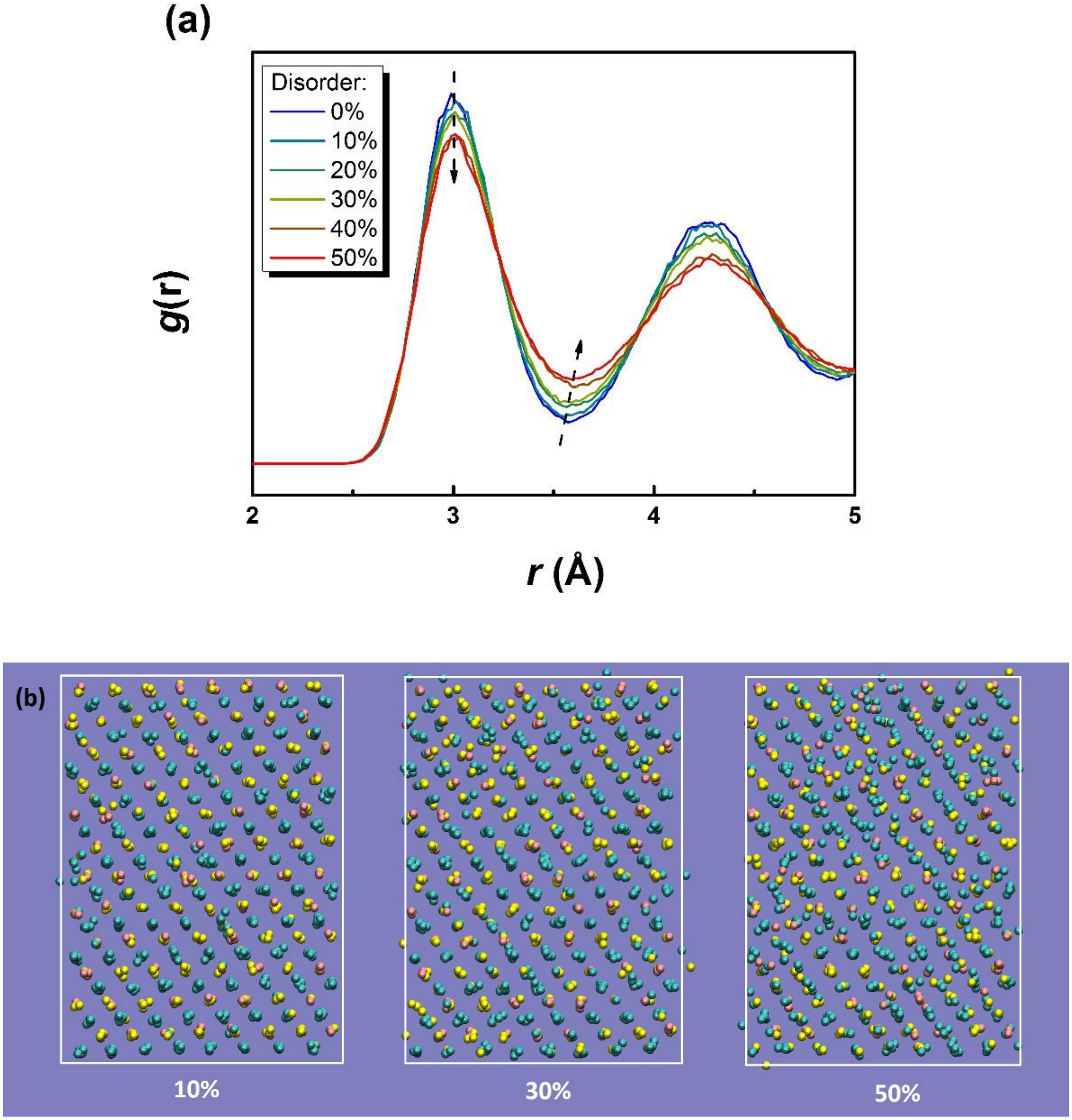


Figure S2. (a) The PDFs of *c*-GST with different degrees of artificial disorder. The percentage of disorder is calculated by the fraction of ASPs with respect to the total number of Sb atoms. The arrows indicate the broadening of the first peak. (b) The instantaneous structures of *c*-GST after 15 ps of AIMD simulations with 10%, 30% and 50% ASPs.

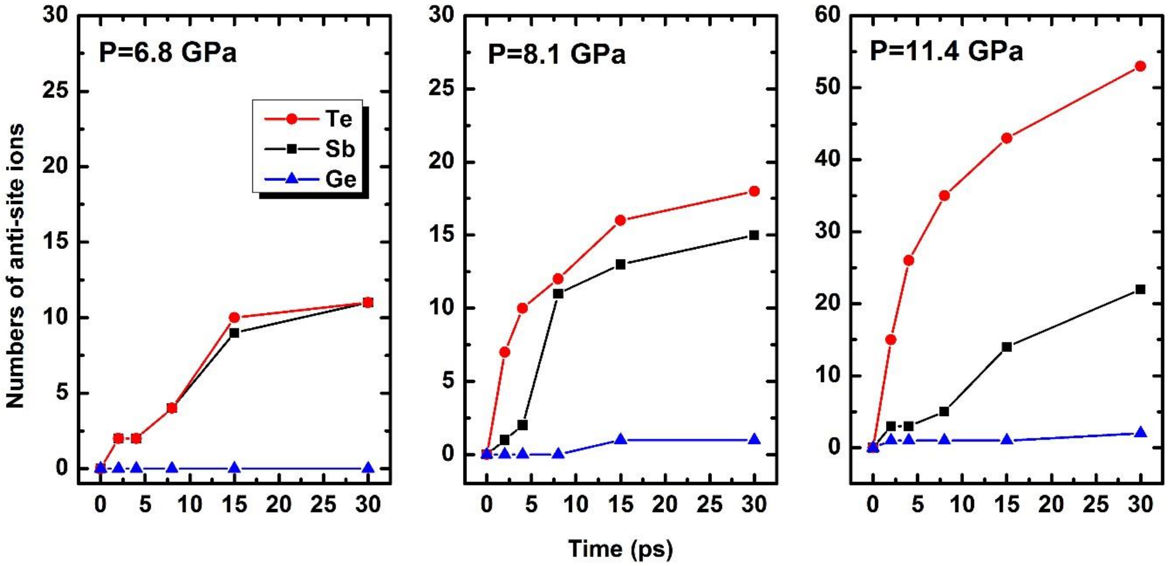


Figure S3. The numbers of anti-site ions evolving with AIMD time. The number of single Te hoppings (without the assistance of Sb) can be calculated roughly by the difference between the numbers of anti-site Sb and Te atoms. At P=6.8 GPa, the anti-site Sb and Te hoppings are well-synchronized and the single Te hopping is not favored. At P=8.1 GPa, single Te hoppings become relevant, and even prominent at P=11.4 GPa.

Table S1. The parameters (temperature, pressure, time) of our simulations and the number of ASPs counted at the end of simulation time. (Total number of atoms in a supercell: 1008)

| Pre-set temperature | Pressure | time | No. of ASPs (at the end of simulation time) |
|---|---|---|---|
| **300K** | 5.4 GPa | 80 ps | 2 |
| **300K** | 6.8 GPa | 80 ps | 2 |
| **300K** | 13.5 GPa | 80 ps | Severally distorted |
| **300K** | 21.4 GPa | 80 ps | Amorphized |
| **450K** | 5.3 GPa | 60 ps | 2 |
| **450K** | 6.8 GPa | 60 ps | 7 |
| **450K** | 13.2 GPa | 60 ps | Severally distorted |
| **450K** | 21.4 GPa | 60 ps | Amorphized |
| **600K** | 0.2 GPa | 100 ps | 1 |
| **600K** | 1.8 GPa | 100 ps | 2 |
| **600K** | 3.0 GPa | 100 ps | 1 |
| **600K** | 5.5 GPa | 100 ps | 7 |
| **600K** | 6.8 GPa | 100 ps | 12 |
| **600K** | 8.1 GPa | 100 ps | 20 |
| **600K** | 11.4 GPa | 100 ps | 28 |
| **600K** | 13.3 GPa | 100 ps | Severally distorted |
| **600K** | 21.8 GPa | 100 ps | Amorphized |
